# Investigating the Current Status of SARS-CoV-2 Antibodies in Hospital Staff

**DOI:** 10.3390/pathogens12050688

**Published:** 2023-05-08

**Authors:** Keh-Sen Liu, Yu-Ying Yang, Kai-Lin Hwang, Hsing-Ju Wu

**Affiliations:** 1 Division of Infectious Diseases, Department of Internal Medicine, Show Chwan Memorial Hospital, Changhua 500, Taiwan; adm630@show.org.tw; 2 Department of Laboratory Medicine, Show Chwan Memorial Hospital, Changhua 500, Taiwan; al009@show.org.tw; 3 Department of Health Business Administration, Hungkuang University, Taichung 433, Taiwan; kai_linh@hotmail.com; 4 Research Assistant Center, Show Chwan Memorial Hospital, Changhua 500, Taiwan; 5 Department of Medical Research, Chang Bing Show Chwan Memorial Hospital, Lukang Township, Changhua County 505, Taiwan; 6 Department of Nursing, Chung-Jen Junior College of Nursing, Health Sciences and Management, Da-Lin Township, Chiayi County 622, Taiwan

**Keywords:** COVID-19, SARS-CoV-2, healthcare worker, antibody, vaccination

## Abstract

The coronavirus disease 2019 (COVID-19) pandemic caused by SARS-CoV-2 had reported over 676 million cases by March 2023. The main aim of this study is to investigate whether the levels of anti-S and anti-N antibodies could precisely indicate the degree of protection against SARS-CoV-2 and affect the probability or time of contracting COVID-19. In this study, a serosurveillance study was conducted in healthcare workers (HCWs) at a regional hospital in Taiwan to evaluate their antibody levels based on infection and vaccination status. Of 245 HCWs enrolled, all have been vaccinated prior to infection. Of these, 85 participants were infected by SARS-CoV-2, while 160 participants were not infected at the time of blood sample collection. The level of anti-SARS-CoV-2 S antibody was significantly higher in the infected HCWs than in the non-infected participants (*p* < 0.001). It is worth noting that the mean duration between the administration of the last dose of the vaccine and the occurrence of SARS-CoV-2 infection was 5.61 ± 2.95 months. Our follow-up survey revealed that the non-infected group had significantly higher levels of antibodies compared to the infected group (all *p* < 0.001). In conclusion, this study suggests that the level of antibodies could serve as a reflection of the protective efficacy against SARS-CoV-2 infection. It has the implication for vaccine decision-making policies in the future.

## 1. Introduction

As of March 2023, the coronavirus disease 2019 (COVID-19) pandemic caused by severe acute respiratory syndrome coronavirus 2 (SARS-CoV-2) had reported over 676 million confirmed cases and 6 million deaths worldwide [1]. In Taiwan, by March 2023, over 10 million cases had been recorded [2], with its first significant wave occurring from May to August 2021 due to the alpha variant [3]. Healthcare workers (HCWs) are at an increased risk of viral exposure due to prolonged contact with infected patients and aerosol generation in the medical environment [4].

The infectious virion of the SARS-CoV-2 virus contains four structural proteins: envelope protein (E), matrix protein (M), spike protein (S), and nucleocapsid protein (N). Only the N and S proteins have been observed to induce high levels of antibodies in patients who were infected naturally. Nevertheless, it is unlikely that antibodies to N can effectively neutralize SARS-CoV-2 [5]. Therefore, the majority of COVID-19 vaccines prioritize the generation of T cells that produce neutralizing antibodies against the spike protein, which contains the receptor binding domain (RBD) [5,6].

To curb the spread of the disease, many countries have developed vaccines to provide herd immunity [7]. Several vaccines have been proven effective against COVID-19, including ChAdOx1 (AstraZeneca, AZ), BNT162b2 (Pfizer), and mRNA1273 (Moderna) [8]. Taiwan’s vaccination program started in March 2021, with most HCWs receiving two doses of the ChAdOx1 vaccine [3]. As additional vaccines became available in Taiwan, Moderna and BNT162b2 were also administrated to HCWs [9]. In July 2021, Taiwan Food and Drug Administration authorized the emergency use of MVC-COV1901, a CpG1018 and aluminum hydroxide-adjuvanted SARS-CoV-2 pre-fusion-stabilized spike protein S-2P vaccine developed by Medigen Vaccine Biologics Corporation. MVC-COV1901 is one of two vaccines included in the WHO Solidarity Trial [3].

The question of passive immunity has arisen despite efforts to maximize vaccine uptake and coverage. Studies have shown that active immunization does not necessarily produce antibodies, and in some cases, the antibody count drops several months after completing the vaccination schedule [10,11]. Terms such as infection-induced immunity, vaccine-induced immunity, and hybrid immunity have been introduced [12]. Neutralizing antibodies are a crucial tool in monitoring an individual’s immune reaction to both vaccination and infection [13]. Assays that measure antibodies against the nucleocapsid protein have demonstrated high sensitivity and specificity in many studies [14]. The first studies show the antibody response after a single dose of SARS-CoV-2 vaccine [15,16].

It is also worth noting that the majority of the studies investigating the levels of SARS-CoV-2 antibodies in HCW were conducted in the Americas or Europe. There was also a lack of data in certain WHO regions, namely the Eastern Mediterranean Region, South-East Asia region and the African region where resources are lower. More prevalence studies need to be conducted in these areas in order to properly estimate the overall seroprevalence of SARS-CoV-2 antibodies in the population. The extent of vaccine-induced protection varies greatly when combined with SARSCoV-2 infections [17], and the clinical implications of immunogenicity for hospital staff are still unclear. Therefore, the main aim of this study is to investigate whether the levels of anti-S and anti-N antibodies could precisely indicate the degree of protection against SARS-CoV-2 and affect the probability or time of contracting COVID-19. To address this, a serosurveillance study was conducted in HCWs at a regional hospital in Taiwan to evaluate their antibody levels based on infection and vaccination status, as well as the different vaccine brands used.

## 2. Material and Methods

### 2.1. Study Design and Participants

The HCWs who had been vaccinated were invited to participate in this prospective study in August 2022. In addition, the hospital staff aged 20~70 years who were not infected with SARS-CoV-2 or had been infected with SARS-CoV-2 were enrolled. The status of infection was determined at the time of blood sample collection. The clinical data and sera of hospital staff in a Regional Hospital in Central Taiwan were collected in this study. A follow-up survey on the infection status of the participants was conducted in January 2023.

### 2.2. Serological Tests

10 mL of blood sample was collected from each participant and serum was separated for anti-N and anti-S antibody serological tests. The humoral immunity response of the vaccines was examined by measuring total immunoglobulin levels to the RBD of the SARS-CoV-2 spike protein using the anti-SARS-CoV-2 S enzyme immunoassay (Elecsys, Roche Diagnostics International Ltd., Mannheim, Germany), according to the manufacturer’s instructions. The assay result ranged from 0.8 to 250 U/mL. The samples with results <0.8 U/mL were regarded as negative for anti-SARS-CoV-2 S. Following vaccination, antibodies against the SARS-CoV-2 N protein were determined using the Elecsys Anti-SARS-CoV-2 assay (Roche Diagnostics, Mannheim, Germany). The assay results were interpreted as nonreactive/negative (cutoff index < 1) and reactive/positive (cutoff index ≥ 1). The pre-existing SARS-CoV-2 infection was defined as positivity for anti-nucleocapsid at any point, anti-spike before vaccination, and/or a history of positive PCR results on the nasopharyngeal swab [18,19].

### 2.3. Statistical Analysis

The interval between the time of sample obtained and the last dose of vaccination was calculated and grouped into three groups: <3 months, ≥3~<6 months, and ≥6 months. The mean, median, standard deviation and quartiles of the interval between the first-time infection date and the last dose of vaccination were presented. Fisher’s exact test was performed to compare the gender, vaccination status, and the combination of vaccine brand for the first three doses between the infected and non-infected individuals. The independent 2-sample *t*-test was used to compare the distribution of age, antibody titer, laboratory assessments, the interval between the time of laboratory testing and the last dose of vaccination, and the interval between the laboratory testing and the subsequent infections between the two groups. Pearson correlation coefficients were calculated to examine the linear associations between time from the last dose of vaccination and SARS-CoV-2 S/N antibodies with natural logarithm transformation for non-infected individuals and various combinations of the vaccine brands. The interval between the date of a positive test and the last dose of the vaccination, and the interval between the laboratory testing and the subsequent infections were calculated and the association between the intervals and SARS-CoV-2 S/N antibodies with/without natural logarithm transformation were also evaluated using the Pearson correlation coefficient for infected HCWs. All statistical analyses were performed using SPSS Version 18.0 for Windows (IBM SPSS Statistics, Chicago, IL, USA) and the level of significance was set at 0.05.

## 3. Results

### 3.1. Demographic Data

Of 245 HCWs enrolled, all have been vaccinated prior to infection. Of these, 85 participants were infected by SARS-CoV-2, while 160 participants were not infected at the time of blood sample collection (Table 1). The mean ages were 37.5 ± 10.2 and 37.1 ± 9.3 years old for the non-infected and infected participants, respectively. Most participants for both groups were female (90.6% and 95.3% for the non-infected and infected participants, respectively) and were predominantly nurses. All of them were vaccinated by 2, 3 or 4 doses of different brands or different combinations. Within each of the two groups, only one participant had received 2 vaccine doses. In the non-infected group, the majority of individuals received 4 doses for <3 months (49.4%, Table 1), and most HCWs received the vaccine combination of 2 AZ + 2 Moderna (*n* = 63, 39.4%). In contrast, in the infected group, most people received 3 doses for ≥6 months (82.4%, Table 1), and the majority of HCWs received a combination of 2 AZ + Moderna (*n* = 56, 65.9%). There were no significant differences in age, sex, vaccination status and the combination of vaccine brands between the infected and non-infected participants (Table 1).

### 3.2. Antibody Level and Other Factor Analysis

Our results revealed that the levels of anti-SARS-CoV-2 S antibodies were significantly higher in the infected HCWs than in those in the non-infected participants (27,885.05 U/mL vs. 11,488.69 U/mL, *p* < 0.001, Table 2). The infected HCWs also showed significantly higher levels of anti-SARS-CoV-2 N antibodies than the non-infected participants (29.93 COI vs. 0.38 COI, *p* < 0.001, Table 2). The mean interval between the last dose of vaccine and the time when the antibody levels were measured in this study were 4.04 months and 6.24 months for the non-infected and infected groups, respectively (Table 2).

It is worthwhile to note that the levels of white blood cells (WBC) were significantly higher in the non-infected participants than those in the infected HCWs (6771.50/μL vs. 6305.65/μL, *p* = 0.050, Table 2), whereas the levels of cholesterol (184.01 mg/dL vs. 194.12 mg/dL, *p* = 0.014) and high-density lipoprotein cholesterol (HDL-C) (62.93 mg/dL vs. 68.04 mg/dL, *p* = 0.008) for the non-infected participants were significantly lower than those for the infected HCWs.

### 3.3. Correlation of Antibody Level and Vaccination or Infection

**Figure 1:**
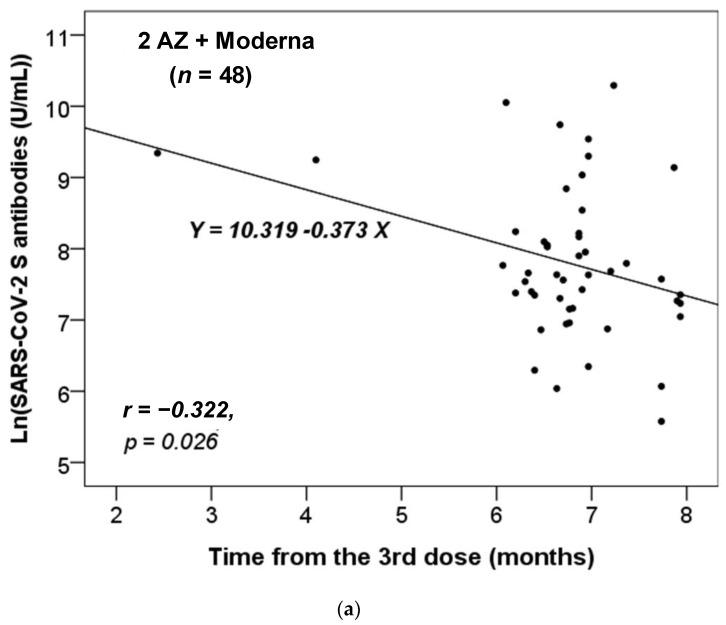

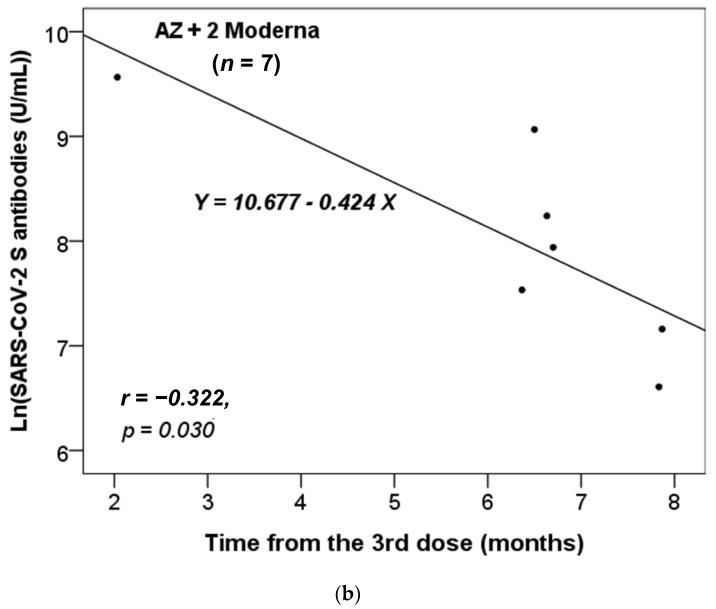
The correlation between the time interval from the last administrated vaccine dose to the time of antibody testing and the level of anti-SARS-CoV-2 S antibody in the non-infected cases vaccinated with the combinations of (**a**) 2 AZ + 1 Moderna and (**b**) AZ + 2 Moderna. Pearson correlation coefficient (*r*) and *p* value were indicated.

We further assessed whether there was a correlation between the time interval from the last administrated vaccine dose to the time of antibody testing and the titers of the anti-SARS-CoV-2 S antibody. We stratified these participants based on the different combinations of vaccines received. As there was only one participant who had received two vaccine doses in each of the non-infected and infected groups (Table 1), we focused our analysis on those who had received three or four doses. Additionally, we only analyzed the results for the vaccine combinations received by more than five participants. Figure 1 shows that only the anti-S antibody levels in the non-infected group received the vaccine combinations of 2 AZ + 1 Moderna (*n* = 48) or AZ + 2 Moderna (*n* = 7) were significantly and inversely correlated with the time from the third dose of vaccination. Thus, the concentration of anti-S antibody level decreased significantly over time from the last dose (*p* = 0.023 and 0.030, respectively).

**Figure 2:**
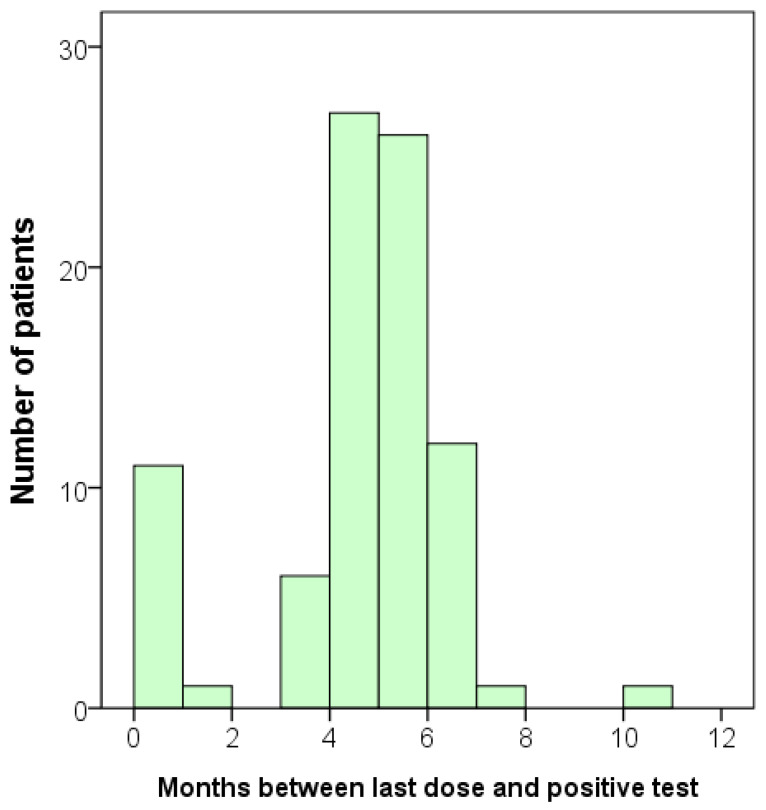
Bar graph displaying the duration between the last dose of vaccination and the time of infection for each participant. Total *n* = 85.

In the infected group, we also investigated whether there was a correlation between the interval from the onset of infection to the time of antibody testing and the concentrations of anti-SARS-CoV-2 S and N antibodies. Again, we concentrated our analysis on those who had received three or four vaccine doses. None of the vaccine combinations showed a significant correlation. However, since all the infected individuals were also vaccinated, it is challenging to observe any trend. Although we did not observe any correlation between the onset of infection and the antibody levels, we did identify a trend that most participants became infected after five or six months following vaccination (Figure 2).

### 3.4. A Follow-Up Survey on the Infection Status

After measuring the concentrations of antibodies, we conducted a follow-up survey on the infection status of the participants. An additional total of 79 participants were infected, including one with a second infection. When we combined the initial ‘infected’ group with the newly identified positive cases, the total number of infected participants increased to 163 (Table 3). Consequently, we obtained additional data that allowed us to analyze the relationship between vaccination and the time of infection or the antibody level in greater detail. The mean duration between the administration of the last dose of the vaccine and the occurrence of SARS-CoV-2 infection was 5.61 ± 2.95 months, with a wide range of 0.03 to 16.47 months (Table 3). This follow-up result was consistent with the findings shown in Figure 2.

Next, we examined the correlation between the level of anti-SARS-CoV-2 S or N antibody and the likelihood of future infection. Our analysis revealed that neither of the anti-S and anti-N antibody levels were significantly correlated with the duration between the time measuring the antibody level and a positive infection (Table 4).

As there was no significant correlation between the levels of antibodies and the occurrence of infection, we proceeded to investigate whether the levels of antibodies could predict the likelihood of future infection. Therefore, we compared the levels of antibodies in participants who remained uninfected (*n* = 166) with those who became infected (*n* = 79) after the measurement of antibodies. Our analysis revealed a significant difference between these two groups. The non-infected group had significantly higher levels of antibodies compared to the infected group (*p* < 0.001, Table 5).

## 4. Discussion

In this research, we assessed the present state of SARS-CoV-2 antibodies among hospital staff and its impact on the degree of protection against SARS-CoV-2 and affect the probability or time of contracting COVID-19. All hospital staff at our facility were vaccinated as they belong to high-risk groups for COVID-19. The anti-S antibodies were higher in infected HCWs compared to non-infected participants (Table 2). The low anti-N antibody response following vaccination in the non-infected group has been noted as expected. It is known that the level of anti-N antibody is not indicative of the individual’s vaccination status, but rather their infection status [18,20]. Moreover, the combination of infection and vaccination significantly increased the levels of anti-SARS-CoV-2 S and anti-SARS-CoV-2 N antibodies (*p* < 0.001, Table 2). Prior evidence has shown that a previous SARS-CoV-2 infection before primary vaccination can efficiently prime individuals for the COVID-19 vaccination [21,22,23]. Moreover, individuals who recovered from COVID-19 and received a dose of the mRNA vaccine showed an increase in all components of the humoral reaction. Bone marrow plasma cells (BMPCs) expressing specific antibodies are long-lasting and have serum neutralizing activity against new variants of concern. These BMPCs are cleared and produced extensively after vaccination, suggesting that immunity in convalescent persons will be long-lasting [24].

Our data revealed that the titer of the anti-S antibody in the non-infected group with only the vaccine combinations of 2 AZ + 1 Moderna and AZ + 2 Moderna decreased over time (Figure 1). This suggests that the protective effects of vaccination combinations of AZ and Moderna may wane over time. Furthermore, our data showed that most participants became infected after five or six months of vaccination (Table 2 and Table 3, and Figure 2), which is consistent with previous studies [25,26]. According to the study by Matusali G. et al. [27], the levels of anti-RBD IgG and anti-trimeric S IgG persisted for up to six months following the second dose of the COVID-19 vaccine. However, the anti-RBD IgG antibodies showed a more rapid decline compared to the anti-trimeric S IgG antibodies [27]. Despite observing continuous seropositivity beyond six months following vaccination, Sarrigeorgiou et al. [28] noted a gradual decline in antibody levels. Thus, our results are consistent with numerous global studies, indicating that racial variances have no substantial impact on the humoral response to vaccines. The immune response following COVID-19 vaccination involves two processes: cellular response—which generates diverse T-cell lineages, interferons, and interleukins—and the generation of IgG antibodies that target viral antigens, including protein S, triggered by the first process [29]. Generally, these antibodies remain detectable for a period of approximately six months, after which they decrease by 5 to 10-fold [30,31,32,33]. As a result, it is necessary to administer a booster dose approximately six months after the initial vaccination, especially if new variants are circulating. It is noteworthy that new variants of concern (VOCs) of SARS-CoV-2 have the potential to increase virus transmissibility and/or disease severity, as well as result in diagnostic and/or treatment failures [34,35,36]. Taiwan experienced a COVID-19 outbreak in May 2021 and later in June 2021 due to the emergence of the Alpha-lineage (B.1.1.7) [37]. The first cases of the Delta-lineage B.1.617.2 were later reported by the Taiwan Central Epidemic Command Center (CECC), which then led to community transmission outbreaks [38].

Our results did not show any correlation between the antibody level and infection (Section 3.3 and Table 4). However, several studies have demonstrated that following infection, there is a decrease in serum anti-SARSCoV-2 antibodies. The decline occurs rapidly in the first 120 days after infection, followed by a slower decline in the subsequent 210 days. Nevertheless, significant antibody levels are maintained for at least 11 months after infection [39,40,41]. S-specific anti-SARS-CoV-2 bone marrow plasma cells (BMPC) were detected in bone marrow aspirates obtained approximately 7 and 11 months post-infection [42]. A correlation was found between the concentration of anti-S IgG BMPC present in bone marrow aspirate and the circulating anti-S IgG titers at 210–240 days after symptom onset in convalescent individuals [43].

There is a need for immunologic surveillance and follow-up among health personnel, particularly in high-risk groups. This surveillance can provide evidence-based data to determine the appropriate timing for administering a booster shot to sustain heightened levels of antibodies and supply enhanced protection against SARS-CoV-2 and its mutated strains [44]. Our research suggests that the quantity of antibodies present may serve as an indicator of protection against SARS-CoV-2 infection (Table 5). These findings are crucial in making decisions regarding vaccination policy in the future. For instance, hospital staff could receive vaccinations every six months, and those with high levels of antibodies against SARS-CoV-2 may not require frequent vaccinations, such as once a year. Therefore, it may be more prudent to assess antibody levels prior to determining the optimal vaccination frequency. These results may inform policymakers, researchers, and clinicians regarding the optimal time to administer booster shots. Our study provides important insights into individual antibody responses to vaccination/infection that can aid clinicians in identifying populations that may require follow-up immunization.

In addition to these findings, non-infected participants had significantly higher levels of WBC than infected HCWs (*p* = 0.050, Table 2), while cholesterol (*p* = 0.014) and HDL-C (*p* = 0.008) levels were significantly lower in non-infected participants compared to infected HCWs. These results suggest a potential association between COVID-19 infection and dyslipidemia. Similarly, a study by Tang et al. [45] proved that high BMI or a low Adp:Lep, indicative of adipose tissue dysfunction and poor metabolic health, was associated with higher cross-reactive IgG titers, potentially linked to increased disease severity. Following vaccination, obesity was associated with significantly lower antibody levels against RBD from Wuhan Hu-1 and B.1.1.7, but not B.1.351, P.1, or B.1.617.2 [45]. Conversely, analysis of individual dyslipidemia components found that hypertriglyceridemia was associated with lower antibody titers in men after the second and third doses [46]. This finding is in line with the previous research indicating that individuals with obesity may exhibit weaker immune responses to other vaccines, including those for influenza [47]. Dyslipidemia has been linked to a higher risk of severe illness and death from COVID-19 [48]. This could be due to dyslipidemia’s negative impact on the immune system, such as activation of inflammasomes [49], overproduction of the pro-inflammatory cytokine interleukin-6 [50], and reduction in serum immunoglobulin levels [51]. These results provide important insights into the potential factors affecting immune response to COVID-19 infection and vaccination, which could inform clinicians and policymakers in developing personalized strategies for disease prevention and management.

Previous studies also attempted to identify the factors that contribute to the variability in this antibody response. A statistically significant reduction in post-vaccine antibody response was observed among smokers compared to nonsmokers. Among individuals with an antibody titer greater than 250 U/mL, the proportion of nonsmokers was 72.5%, whereas the proportion of smokers in the same group was only 27.5% [52]. Similarly, the study by Moncunill et al. [53] demonstrated that smokers and those with underlying comorbidities, such as autoimmune and other immunological disorders, chronic respiratory and renal diseases, heart and liver diseases, diabetes, and cancers, displayed markedly reduced antibody levels and decreased plasma neutralizing capacity. Yamamoto et al.’s study [54] with a large sample size revealed that both heated tobacco product users and exclusive cigarette smokers had significantly lower titers of anti-S IgG antibodies compared with non-smokers. An evident decline in antibody levels was also noticeable as alcohol consumption increased, even with moderate alcohol intake [54]. This finding is consistent with the study by Kageyama et al. [55]. In addition, Kageyama et al. demonstrated that patients who were taking immunosuppressive drugs or glucocorticoids showed decreased antibody responses. However, medication prescribed for an allergy was found to be a factor significantly correlated with higher levels of antibodies [55]. In contrast, the findings of Wratil et al. suggest that smoking behavior may be protective against the SARS-CoV-2 infection [56].

This study predominantly consisted of females (90.6% and 95.3% for the non-infected and infected participants, respectively) as most participants enrolled were female nurses. Similarly, other studies investigating HCWs also reported higher proportions of female participants [19,57,58]. Several studies have shown that women generally exhibit a more substantial antibody response to vaccine as compared to men [55,57,58,59,60].

There are several limitations to this study that should be noted. Firstly, due to the use of binding antibodies as the only measure, it was not possible to confirm the immunogenicity of the vaccine, such as the T-cell response. Secondly, this study did not involve a real follow-up design, and there were no regular blood tests taken after vaccination to monitor changes in antibody levels over time. Additionally, we did not collect blood samples prior to vaccination. Thirdly, our sample size was small, and there was a gender distribution bias towards female healthcare personnel, and most were nurses. As this study only included HCWs, there were no elderly participants. Finally, since all participants received the vaccine, it was not possible to compare the effects of vaccination and natural infection on antibody levels. Therefore, further studies with larger sample sizes and follow-up designs are necessary.

## 5. Conclusions

In conclusion, we demonstrated that the antibody levels could reflect the protective effectiveness against SARS-CoV-2 infection; the non-infected group exhibited significantly elevated levels of antibodies compared to the infected group. The protective effects of vaccination may wane over time. The mean period from the last vaccine dose to the onset of SARS-CoV-2 infection was 5.61 ± 2.95 months. Therefore, the finding of this study has implications for vaccine decision-making policies in the future. It suggests that hospital staff may benefit from receiving a vaccination every six months and testing their antibody levels against SARS-CoV-2 prior to vaccination. Those with high antibody levels may not require frequent vaccination, such as once a year.

## Figures and Tables

**Table 1:** Demographic data of the participants.

	Non-Infected		Infected		
	*n*	%	*n*	%	*p*-Value
All	160	100.0	85	100.0	
Sex					0.221
Female	145	90.6	81	95.3	
Male	15	9.4	4	4.7	
Age (years)					0.755
mean, SD	37.5	10.2	37.1	9.3	
median, range	36.1	22~63	36.0	22~60	
Vaccination status (doses and time since last dose)					
2 doses					
≥6 months	1	0.6	1	1.2	
3 doses					0.278
<3 months	3	1.9	0	0.0	
≥3~<6 months	4	2.5	2	2.4	
≥6 months	73	45.6	70	82.4	
4 doses					
<3 months	79	49.4	12	14.1	

*p*-value by Fisher’s exact test or 2-independent samples *t*-test when appropriate.

**Table 2:** The amount of anti-SARS-CoV-2 antibodies and other clinical data for the participants.

	Non-Infected		Infected		
	Mean	SD	Mean	SD	*p*-Value
anti-SARS-CoV-2 S antibodies (U/mL)	11,488.69	13,981.17	27,885.05	19,757.79	<0.001
anti-SARS-CoV-2 N antibodies (COI (cut-off index))	0.38	1.94	29.93	24.11	<0.001
WBC (/μL)	6771.50	1771.44	6305.65	1754.66	0.050
Hb (g/dL)	13.12	1.25	12.99	1.57	0.485
PLT (×10^4^/μL)	30.13	6.65	29.85	5.67	0.734
Hct (%)	40.54	3.28	40.24	3.81	0.521
AST (U/L)	17.93	6.33	17.04	4.17	0.190
ALT (U/L)	16.54	12.08	14.61	10.29	0.212
BUN (mg/dL)	11.86	3.67	12.15	3.00	0.531
Cr (mg/dL)	0.68	0.45	0.65	0.13	0.490
Cholesterol (mg/dL)	184.01	28.56	194.12	33.37	0.014
TG (mg/dL)	88.58	48.43	82.83	52.98	0.396
HDL-C (mg/dL)	62.93	13.88	68.04	14.40	0.008
LDL-C (mg/dL)	109.74	26.11	115.46	29.73	0.122
eGFR (mL/min/1.73 m^2^)	116.76	24.80	125.28	89.75	0.262
Glucose (mg/dL)	93.17	18.46	89.33	9.46	0.076
HbA1C (%)	5.44	0.59	5.32	0.33	0.093
Interval between the last vaccine dose and the time of antibody testing (months)	4.04	2.89	6.24	2.12	<0.001

*p*-value 2-independent samples *t*-test.

**Table 3:** Time of infection since the last dose of vaccination.

n	163
Mean, SD	5.61, 2.95
Median, IQR	5.23, 3.20
Range	0.03~16.47

SD: Standard deviation; IQR: Inter-quartile range (75th percentile–25th percentile).

**Table 4:** Correlation between the levels of antibodies and the interval (months) between the time point measuring the antibody level and a positive infection for cases that became infected after the laboratory tests were conducted.

*n* = 79	Pearson Correlation Coefficient	*p*-Value
SARS-CoV-2 S antibodies (U/mL)	−0.089	0.436
SARS-CoV-2 N antibodies (COI (cut-off index))	0.006	0.961
Ln(SARS-CoV-2 S antibodies (U/mL))	−0.130	0.252
Ln(SARS-CoV-2 N antibodies)	−0.043	0.704

**Table 5:** Cases infected versus non-infected after the antibody evaluations.

	Non-Infected (*n* = 166)		Infected * (*n* = 79)		
	Mean	SD	Mean	SD	*p*-Value
SARS-CoV-2 S antibodies (U/mL)	20,616.42	20,176.79	9950.56	8422.74	<0.001
SARS-CoV-2 N antibodies (COI (cut-off index))	15.58	22.72	0.22	0.91	<0.001
Ln(SARS-CoV-2 S antibodies (U/mL))	9.42	1.18	8.70	1.17	<0.001
Ln(SARS-CoV-2 N antibodies)	0.50	2.76	-2.19	0.56	<0.001

* Infections occurred after the laboratory tests; *p*-value by Independent 2-sample *t*-test.

## Data Availability

All data in this study are included in the article.
